# Ultra‐Fast Molecular Rotors within Porous Organic Cages

**DOI:** 10.1002/chem.201704964

**Published:** 2017-11-22

**Authors:** Ashlea R. Hughes, Nick J. Brownbill, Rachel C. Lalek, Michael E. Briggs, Anna G. Slater, Andrew I. Cooper, Frédéric Blanc

**Affiliations:** ^1^ Department of Chemistry University of Liverpool Crown Street Liverpool L69 7ZD UK; ^2^ Materials Innovation Factory University of Liverpool 51 Oxford Street Liverpool L7 3NY UK; ^3^ Stephenson Institute for Renewable Energy University of Liverpool Crown Street Liverpool L69 7ZD UK

**Keywords:** molecular rotors, NMR spectroscopy, porous organic cages, responsive materials, supramolecular chemistry

## Abstract

Using variable temperature ^2^H static NMR spectra and ^13^C spin‐lattice relaxation times (T_1_), we show that two different porous organic cages with tubular architectures are ultra‐fast molecular rotors. The central *para*‐phenylene rings that frame the “windows” to the cage voids display very rapid rotational rates of the order of 1.2–8×10^6^ Hz at 230 K with low activation energy barriers in the 12–18 kJ mol^−1^ range. These cages act as hosts to iodine guest molecules, which dramatically slows down the rotational rates of the phenylene groups (5–10×10^4^ Hz at 230 K), demonstrating potential use in applications that require molecular capture and release.

Porous organic frameworks (POFs) are supramolecular assemblies that have ordered architectures containing an inherent void. Examples include metal organic frameworks (MOFs), covalent organic frameworks (COFs), zeolitic imidazolate frameworks (ZIFs), and porous organic cages (POCs).^1^, ^2^, ^3^, ^4^ POCs differ from these other framework families and consist of discrete molecules containing both an intrinsic void within the cages in addition to voids within the overall lattice; because of these properties, POCs are also solution‐processable and have been explored for a range of applications including catalysis, molecular separation and gas storage.^5^, ^6^, ^7^, ^8^, ^9^, ^10^ For example, POCs have been shown to capture guest molecules such as iodine, SF_6_, and hydrocarbons.^11^ The sequestration of iodine, which is an unwanted fission product,^12^, ^13^, ^14^ is of strong importance for the nuclear industry.^15^ The selective loading and retention of guests such as iodine often relies on molecular flexibility and dynamics, and the understanding of these processes plays a central role in the design of the next generation of POFs.

Recently, a new family of POCs with a chiral, tubular covalent cage (TCC) architecture has been discovered.^10^ These TCCs consist of three “walls” bound by *trans*‐imine cyclohexane linkers. Two of these structures (TCC2‐*R* and TCC3‐*R*) are shown in Figure 1; they differ only by the additional acetylene moieties between the phenylene rings in TCC3‐*R*. The intrinsic pore within these molecules permits guest sorption into the tubular cavities.^4^, ^16^ The static imine cyclohexane linkers and rotating phenylene groups allow these POCs to be classified as molecular rotors.^17^, ^18^, ^19^ It is likely that the window dynamics in these molecules control guest loading into the molecular pore, and we therefore set out to understand the response of these cages to external stimuli, such as guest inclusion.

**Figure 1:**
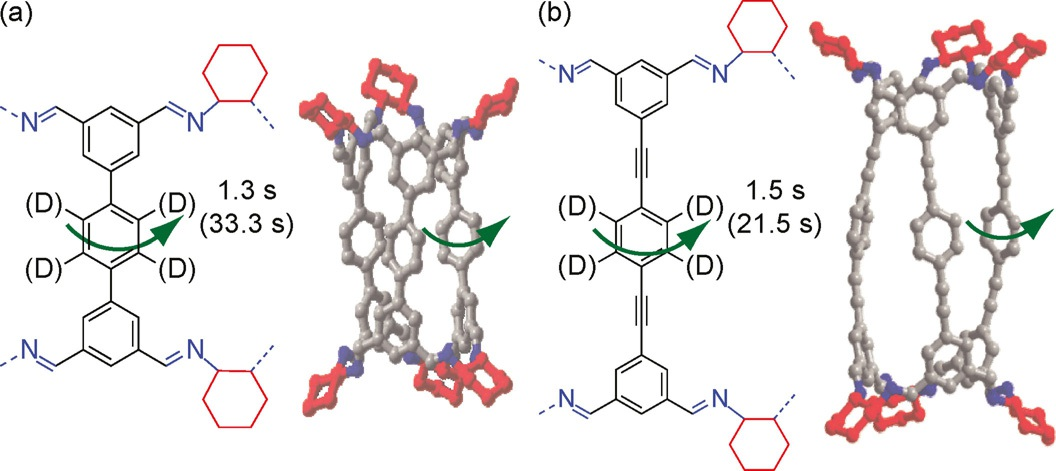
Chemical structure and side view of the X‐ray crystal structure of (a) TCC2‐*R* and (b) TCC3‐*R*.^10^ The cyclohexane groups are shown in red; other C, grey; N, blue; H omitted for clarity in the crystal structure representation. The green arrows indicate fast molecular rotation of the *para*‐phenylene. The ^13^C spin‐lattice relaxation times (T_1_) obtained for selected carbons are given in the Figure, including T_1_ values after iodine loading (values in parentheses). The deuterium‐labelled positions on the *para*‐phenylene are also shown for [D_12_]TCC2‐*R* and [D_12_]TCC3‐*R*.

^2^H solid echo NMR has become an extremely powerful tool for the understanding of dynamics in the kHz timescale,^20^, ^21^, ^22^ which is enabled by the large change in the ^2^H NMR line shape with temperature. This approach provides a qualitative description of the motion as well as its rate and associated activation energies in both pristine fast molecular rotors and those hampered by guest loading (e.g., H_2_O, acetone, iodine, CH_4_ and hydrocarbons);^23^, ^24^, ^25^, ^26^, ^27^, ^28^, ^29^, ^30^, ^31^, ^32^, ^33^ these include mesoporous *para*‐phenylene silica,^23^ polyaromatic frameworks (PAFs),^24^ and MOFs.^28^, ^29^, ^31^, ^33^

Additionally, ^13^C T_1_ values provide correlation times in the MHz frequencies and complementary details of the molecular reorientation. Here, we performed variable temperature ^2^H solid echo NMR experiments and room temperature ^13^C T_1_ measurements on pristine and iodine‐loaded TCC2‐*R* and TCC3‐*R* materials to understand the rotational dynamics of the cages and their host–guest interactions.

TCC2‐*R* and TCC3‐*R* cages were synthesized using literature procedures.^10^ Cages deuterated on the *para*‐phenylene rings (i.e., [D_12_]TCC2‐*R* and [D_12_]TCC3‐*R*, Figure 1) were prepared via a similar approach^10^ using [D_4_]1,4‐dibromobenzene‐2,3,5,6 and these were used to record the ^2^H NMR data (see the Supporting Information for details of all samples preparation and characterisation). In these experiments, the natural abundance ^2^H signals (0.015 %) from the other hydrogens in the cyclohexane linkers and trisubstituted benzene rings are not detected and the ^2^H NMR spectra only probe the dynamics of the *para*‐phenylene moieties of the cages.

^2^H static echo NMR spectra in the 105–298 K temperature range were recorded on desolvated and iodine‐loaded [D_12_]TCC2‐*R* and [D_12_]TCC3‐*R* cages (Figure 2). With decreasing temperature a gradual change in the ^2^H NMR line shape is observed for these materials, however, the motion induced T_2_ anisotropy is dependent on the particular cage. Line shape simulations of the ^2^H NMR spectra support a motion consisting of a rapid two‐site 180° flip reorientation of the *para*‐phenylene ring along its *para* axis, and this provides the rate of molecular reorientation (*k*) at each temperature on the kHz timescale.^19^, ^34^, ^35^

**Figure 2:**
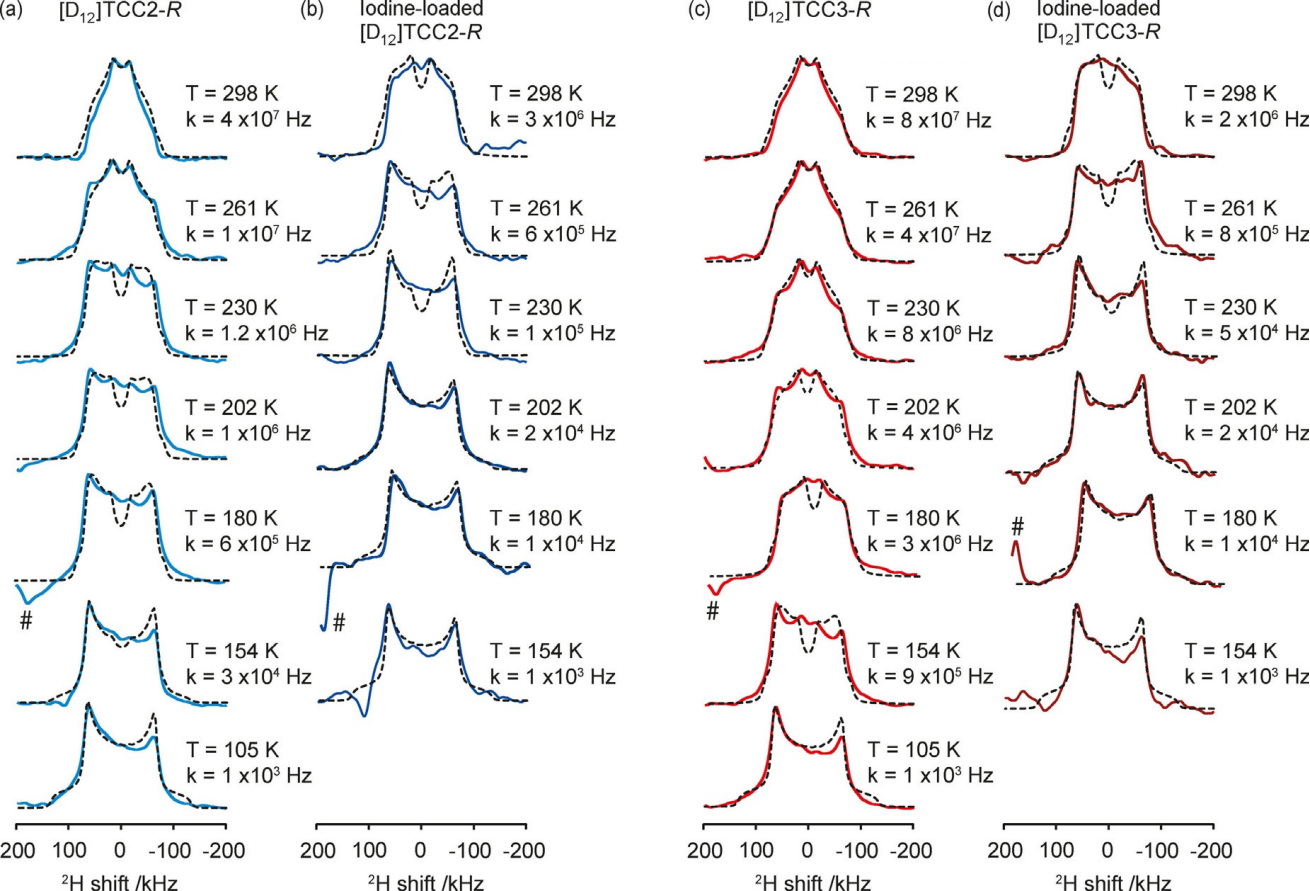
^2^H static solid echo NMR spectra of (a) [D_12_]TCC2‐*R* (blue), (b) iodine‐loaded [D_12_]TCC2‐*R* (dark blue), (c) [D_12_]TCC3‐*R* (red), (d) iodine‐loaded [D_12_]TCC3‐*R* (burgundy) and their corresponding simulated spectra (black dashed lines) obtained at various temperatures. The rotational rates, *k*, obtained from numerical simulations of the NMR lineshapes are also given. Spectral artefacts are denoted with (#).

Figure 2 (a) and (c) show the variable temperature evolution of the ^2^H static echo NMR spectra of [D_12_]TCC2‐*R* and [D_12_]TCC3‐*R* cages. As the temperature decreases from 298 to 105 K, the appearance of the outside horns around ±60 kHz agrees with slower rotation rates at low temperature and a static motional regime at 105 K with the anticipated Pake doublet pattern. The line shape evolution of both cages is analogous, as anticipated for materials with similar tubular covalent architectures. However, the ^2^H NMR spectra of [D_12_]TCC3‐*R* suggest a significantly larger jump rate, as evidenced by the weakening of the spectral shoulders of this cage at a lower temperature (230 K) compared to [D_12_]TCC2‐*R* (261 K), showing slower motion for the latter.

Rotational rates of around 1.2×10^6^ and 8×10^6^ Hz were extracted for [D_12_]TCC2‐*R* and [D_12_]TCC3‐*R* cages, respectively, at 230 K. These data suggest that the presence of an acetylene group enables easier rotation of the *para*‐phenylene ring in [D_12_]TCC3‐*R* by reducing the strong steric interactions of the *ortho*‐hydrogens and opening up the void space (Figure 1). Additionally, it is also possible that small electronic factors play a role. For example, the smaller degree of conjugation between the adjacent phenylene rings in [D_12_]TCC3‐*R* and [D_12_]TCC2‐*R* likely contributes to the smaller activation barrier seen for *para*‐phenylene rotation in the former.

Moreover, while the [D_12_]TCC2‐*R* rate is comparable to other organic frameworks,^27^, ^28^, ^29^, ^36^ the very fast reorientation rate value obtained for the [D_12_]TCC3‐*R* is larger than in any exclusively organic systems reported previously below ≈200 K (Table S3).^18^, ^19^, ^24^, ^29^, ^32^, ^37^, ^38^, ^39^ In particular, below this temperature, the dynamics of [D_12_]TCC3‐*R* are faster than the ones observed very recently for the *para*‐phenylene reorientation in bis(sulfophenylethynyl)‐benzene frameworks based on an overall similar architecture of a phenylene molecular rotor sandwiched between two acetylene moieties (Figure 1 (b)), which previously showed the largest reorientation rate for porous organic materials to date.^32^

At temperatures higher than 298 K, the ^2^H NMR lineshape of the [D_12_]TCC2‐*R* and [D_12_]TCC3‐*R* pristine cages is characteristic of that of the fast motional regime with rates exceeding 10^8^ Hz (Figure S5 (a) and (c)). No additional change in lineshape occurs at higher temperature probably indicating an absence of C−D librational motion and is in sharp contrast to what is observed in PAFs.^24^ This difference likely arises from the more flexible nature of the PAFs architecture compared to the relative rigidity of these TCC2‐*R* and TCC3‐*R* cage structures (Figure 1).

A plot of ln *k* as a function of reciprocal temperature *T* ^−1^ (Figure 3) shows a linear Arrhenius behaviour from which rotational activation energies, *E*_a_, were obtained (Table 1), along with extrapolated rotational rates at infinite temperature (attempt frequencies), *k*_0_, of (9±4)×10^10^ and (10±7)×10^9^ Hz for [D_12_]TCC2‐*R* and [D_12_]TCC3‐*R*, respectively. The larger *E*_a_ value obtained for [D_12_]TCC2‐*R* versus [D_12_]TCC3‐*R* is again consistent with stronger steric interactions in the terphenylene cage structure. The *k*_0_ values obtained are on the low side of the ≈10^12^ Hz^22^ value often associated with *para*‐phenylene rotation, although these values vary significantly with the systems studied and *k*_0_ in the 10^8^–10^41^ Hz range are known.^19^, ^23^, ^24^, ^27^, ^28^, ^29^, ^32^, ^36^, ^37^, ^40^, ^41^ The associated change in entropy (Δ*S*) is negative and is tentatively assigned to correlated rotational motion (Table S2).^41^, ^42^

**Figure 3:**
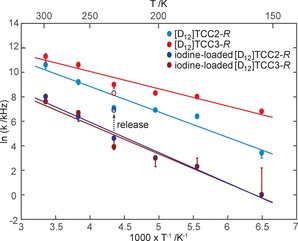
Arrhenius plot of the rotational rates, k, of the *para*‐phenylene ring in desolvated and iodine‐loaded [D_12_]TCC2‐*R* and [D_12_]TCC3‐*R* molecular rotor cages. The lines show the linear fit to the Arrhenius equation with the extracted values being reported in Table 1. Error bars are estimated from comparison of the ^2^H NMR line shape fit at various rotational rates. The errors associated with the 154 K rates for iodine‐loaded [D_12_]TCC2‐*R* and [D_12_]TCC3‐*R* are due to the indistinguishable line shape between 1 and 9 kHz. The ^2^H NMR rotational rates obtained at 230 K from the iodine released [D_12_]TCC2‐*R* and [D_12_]TCC3‐*R* cages are also shown in empty circles.

**Table 1:** Comparison of the activation energy barriers (*E*_a_) and rotational rates at 230 K (*k*_230K_) for all the TCC cages investigated.

Tubular covalent cages	*E*_a_ [kJ mol^−1^]^[a]^	*k*_230_ [K Hz^−1^]
[D_12_]TCC2‐*R*	18 (18–20)	1.2×10^6^
iodine‐loaded [D_12_]TCC2‐*R*	21 (15–21)	1×10^5^
[D_12_]TCC3‐*R*	12 (10–13)	8×10^6^
iodine‐loaded [D_12_]TCC3‐*R*	21 (14–21)	5×10^4^

[a] Range of *E*_a_ values estimated from errors in the values of *k* are given in brackets.

Iodine was loaded into [D_12_]TCC2‐*R* and [D_12_]TCC3‐*R* using a chemical vapour sublimation procedure (Supporting Information) to determine if guest addition hampers motional dynamics in these systems.^23^, ^24^ Upon exposure to iodine at room temperature, the colour of the cages changed from yellow to black, and the guest uptake was monitored gravimetrically (Supporting Information). This revealed a high loading of 10 and 12 iodine atoms per TCC2‐*R* and TCC3‐*R* cage molecule, respectively, after 40 hours of iodine exposure. The variable temperature ^2^H static echo NMR spectra of iodine‐loaded [D_12_]TCC2‐*R* and [D_12_]TCC3‐*R* are given in Figures 2 (b) and (d). There is a clear difference in rotational rates of the molecular rotor between the empty and guest‐loaded materials. The less rapid two‐site ring flip is particularly evident at 230 K for the iodine‐loaded materials, reducing the rotational rates to only 10^5^ and 5×10^4^ Hz for [D_12_]TCC2‐*R* and [D_12_]TCC3‐*R*, respectively (Table 1). This is smaller than the change seen when iodine is loaded into other porous materials,^24^ which probably relates to the different host–guest properties of the materials.

When iodine‐loaded TCC2‐*R* and TCC3‐*R* are heated above room temperature, almost complete release of iodine is observed as detected by thermogravimetric analysis (Supporting Information) and visual inspection of the sample; as a result, large reorientation rates are obtained in the corresponding ^2^H static echo NMR spectra (Figures S6 and S7) and highlight again that these materials are responsive with the rotational dynamics being modulated by the capture and release of a guest molecule.

Additionally, Arrhenius plots yield an increase of *E*_a_ with respect to the guest‐free cages: from 18 to 21 kJ mol^−1^ for [D_12_]TCC2‐*R* and from 12 to 21 kJ mol^−1^ for [D_12_]TCC3‐*R* (Table 1). These data show that the presence of the iodine guest within the void of the cages, and potentially in extrinsic voids between cages, hampers but does not totally suppress molecular reorientation of the *para*‐phenylene rings. It has been reported that when the motion of a guest molecule is restricted within a cavity, fast librational motions are expected;^43^ this is not apparent in these TCC2‐*R* and TCC3‐*R* cages, since even when a guest is located inside the cages, the lineshapes remain consistent with a 180° site reorientation of the *para*‐phenylene rings.

Finally, ^13^C T_1_ values were also monitored due to their strong dependence on molecular motion on the MHz timescale.^43^ The considerably shorter room temperature ^13^C T_1_ values of 1.3–1.5 s obtained in TCC2‐*R* and TCC3‐*R* (see Figures 1, S1 and Table S1) for the CH carbons on the *para*‐phenylene ring versus the other carbons in the cages (appearing in the range of 4–7 s) suggests an efficient relaxation mechanism and rapid molecular reorientation of the cage windows, supporting the fast molecular rotors of these cages. The ^13^C T_1_ relaxation times were found to increase significantly upon loading (Figure 1, Table S1) and is consistent with the change in ^2^H solid echo NMR line shape is noted above where guest addition into the central void slows reorientation of the phenylene rings within the cage structures.

To conclude, we employed ^2^H solid echo NMR and determined ^13^C T_1_ values to probe the rotational dynamics of the *para*‐phenylene rings that define access to the central void in two porous chiral tubular covalent cages, TCC2‐*R* and TCC3‐*R*. Using ^2^H NMR, we show that TCC2‐*R* cages show reorientation rates that are comparable with the fastest molecular rotors reported for other porous frameworks; TCC3‐*R* cages display even faster dynamics (below 200 K), with a very small activation energy barrier, which is ascribed to the facile rotation around the acetylene bonds, due to a reduction in the steric hindrance present. Iodine loading slows the phenylene rotation considerably, as further supported by the lengthening of the ^13^C T_1_ values, while high temperature treatment induces iodine release and reacceleration of the phenylene rings reorientation rates. These data show that the effect on cage dynamics is highly guest dependent, which might have important implications for processes such as competitive loading, molecular separation, and drug release. These findings also emphasize that models of porosity derived from static single crystal structures might be misleading, but that “time‐averaged” models of the pore space could be equally inappropriate because guest inclusion can switch the rotational dynamics off. This suggests that computational models for loading in such systems need to capture the interplay of guest inclusion and rotational dynamics in the porous host.

## Conflict of interest

The authors declare no conflict of interest.

## Supporting information

As a service to our authors and readers, this journal provides supporting information supplied by the authors. Such materials are peer reviewed and may be re‐organized for online delivery, but are not copy‐edited or typeset. Technical support issues arising from supporting information (other than missing files) should be addressed to the authors.

Supplementary
