# Supplementary material for: Ultra‐Fast Molecular Rotors within Porous Organic Cages
Source: Chemistry. 2017 Nov 22;23(68):17217–21. doi: 10.1002/chem.201704964 (PMC5813266; doi:10.1002/chem.201704964)
Supplement: Supplementary file 1 — Supplementary [file CHEM-23-17217-s001.pdf]

CHEMISTRY
A European Journal

Supporting Information

Ultra-Fast Molecular Rotors within Porous Organic Cages

Ashlea R. Hughes,[a] Nick J. Brownbill,[a] Rachel C. Lalek,[a] Michael E. Briggs,[a, b]
Anna G. Slater,[a, b] Andrew I. Cooper,[a, b] and Frédéric Blanc*[a, c]

chem_201704964_sm_miscellaneous_information.pdf

Table of Contents

S1. Solid State NMR methods **S2**

S2. Powder XRD data **S11**

S3 Thermogravimetric analysis **S12**

S4. Infrared spectroscopy **S13**

S5. Synthesis of the chiral tubular covalent cages **S15**

S6. Rotational rates within the literature. **S21**

S7. References **S22**

S1. Solid State NMR methods

All ^2H and ^{13}C solid-state NMR experiments were performed on a 9.4 T Bruker Avance III spectrometer equipped with a 4 mm HXY Magic Angle Spinning (MAS) probe in double resonance mode. The ^1H channel was tuned to $\nu_0(^1H)$ = 400.13 MHz and the X channel tuned to either $\nu_0(^2H)$ = 61.42 MHz, or $\nu_0(^{13}C)$ = 100.62 MHz. All ^1H-^{13}C cross polarisation (CP) spectra were recorded at ν_r = 12.5 kHz with a ^{13}C rf field of 45 kHz for an optimal contact time of 2 ms, while the ^1H rf field amplitude was ramped to obtain maximum signal at approximately 60 kHz. ^1H decoupling was applied at 83 kHz with a SPINAL-64 shaped pulse.[S1] Typically 1024-3072 scans were accumulated and were recorded with a recycle delay corresponding to the maximum signal to noise per unit time of 1.3 x T_1.[S2] Variable temperature ^2H static solid echo data were obtained using a pulse delay of 30 μs at a ^2H rf field of 65 kHz. Fully relaxed spectra were acquired with a minimum of 512 scans and recycling delays ranging from 2 to 15 s depending on temperature. NMR data were processed with TopSpin 3.2 and MATLAB R2016b.[S3] Temperature calibration was preformed prior to NMR data acquisition using the ^{207}Pb chemical shift of $Pb(NO_3)_2$ according to procedure outlined in the literature.[S4,S5] All temperatures reported are actual sample temperatures and have an estimated accuracy of ± 10 K. ^1H spectra were referenced to H_2O at 4.8 ppm, ^{13}C spectra were referenced to CH of adamantane at 29.45 ppm, corresponding to TMS at 0 ppm,[S6] and ^2H spectra were referenced to D_2O at 0 ppm.

Theoretical simulation of the ^2H solid echo NMR spectra were performed using the Express 3.0 program[S7] supported in MATLAB. A powder average of 1000 was used along with the ZCW tiling algorithm.[S7] A two site jump model was used for the *para*-phenylene ring flip with a β angle of 60° and a γ between 0° and 180°. A probe band width of 300 kHz was also applied. The resulting simulation was then left shifted to the top of the echo and lorentzian broadening applied.

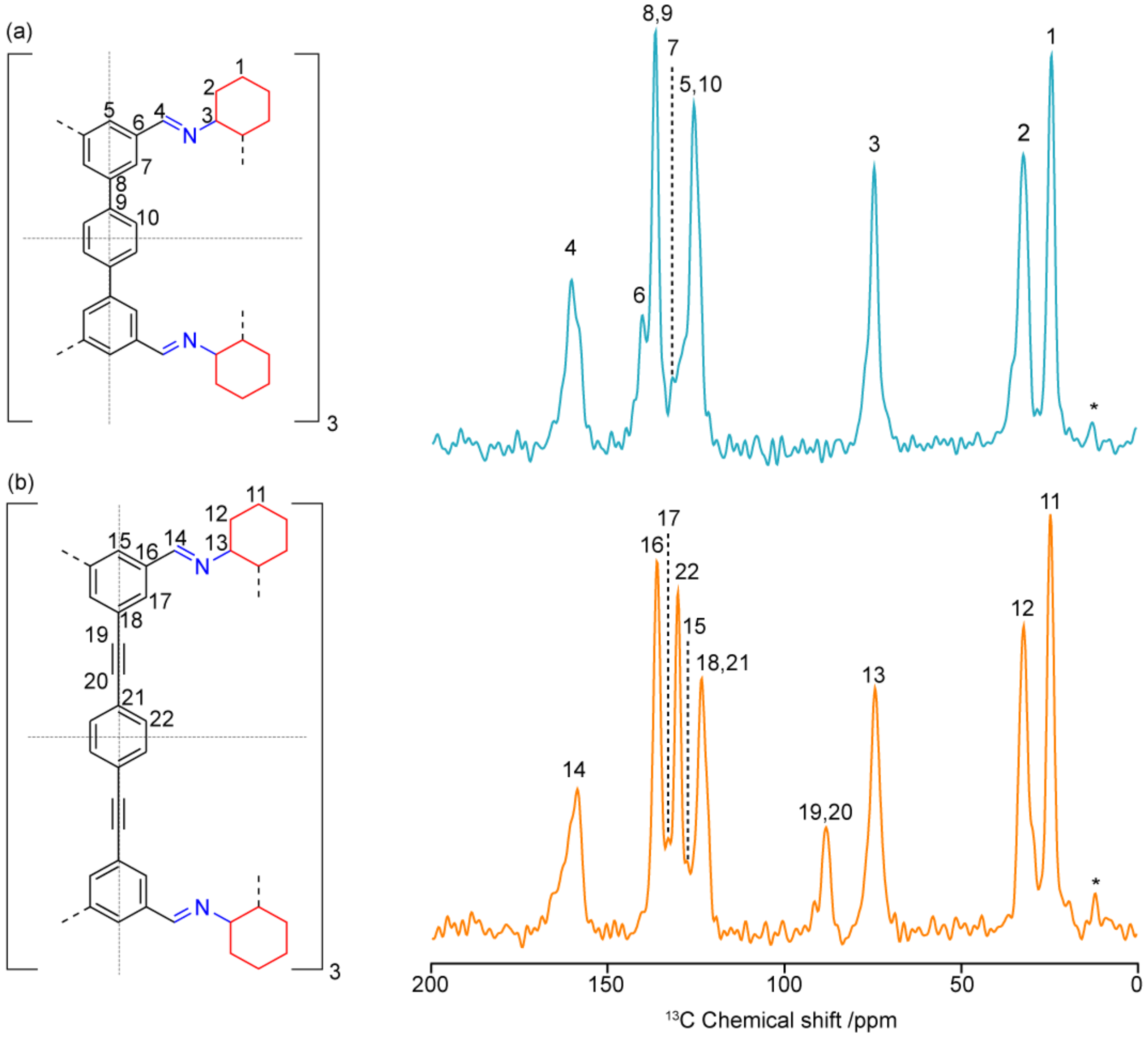

Figure S1. Chemical structure and corresponding ^{13}C CP MAS NMR spectra at ν_r = 12.5 kHz of (a) TCC2-*R* (light blue) and (b) TCC3-*R* (orange). The spectral assignments are given in the figure, with lines of symmetry shown and spinning sidebands marked with asterisks (*).

Table S1. ^{13}C NMR spectra assignments, ^{13}C isotropic chemical shifts, δ, and room temperature ^{13}C spin-lattice relaxation times T_1 obtained for pristine and iodine-loaded TCC2-*R* and TCC3-*R* cages.

Assignments	^{13}C assignment numbers	δ (^{13}C) /ppm	T_1 /s	Iodine-loaded T_1 /s
TCC2-*R*				
-H**C**=N-	4	161	4.8 ± 1.0	32.0 ± 12.1
-**C**HC=N-	6	141	5.9 ± 2.3	63.7 ± 23.8
-**C**CC'H- + -**C**C'H-	8, 9	137	4.9 ± 0.5	52.2 ± 4.7
-HCCCHC**C**- + -**C'**H- + -**C**HCCC'H-	5, 7, 10	126	1.3 ± 0.3	33.3 ± 5.0
-**C**HN-	3	75	5.4 ± 1.0	20.2 ± 8.4
-**C**H_2CHN-	2	33	5.6 ± 0.8	13.9 ± 3.2
-**C**H_2CH_2CHN-	1	25	5.9 ± 0.9	11.5 ± 2.1
TCC3-*R*				
-H**C**=N-	14	161	4.2 ± 0.9	19.7 ± 5.8
-**C**HC=N- + -**C**HCC-	16, 17	137	4.9 ± 0.6	28.0 ± 4.3
-**C**'H-	22	132	1.5 ± 0.3	21.5 ± 2.0
-H**C**CCHCC- + - C**C**CH- + -**C**C'H-	15, 18, 21	125	5.8 ± 0.5	32.2 ± 4.1
-**C**≡CCC'H- + - C≡**C**CC'H-	19, 20	90	7.1 ± 2.5	38.3 ± 7.4
-**C**HN-	13	75	7.5 ± 0.9	31.1 ± 5.2
-**C**H_2CHN-	12	33	7.3 ± 0.5	21.6 ± 2.8
-**C**H_2CH_2CHN-	11	25	7.3 ± 0.5	20.3 ± 2.0

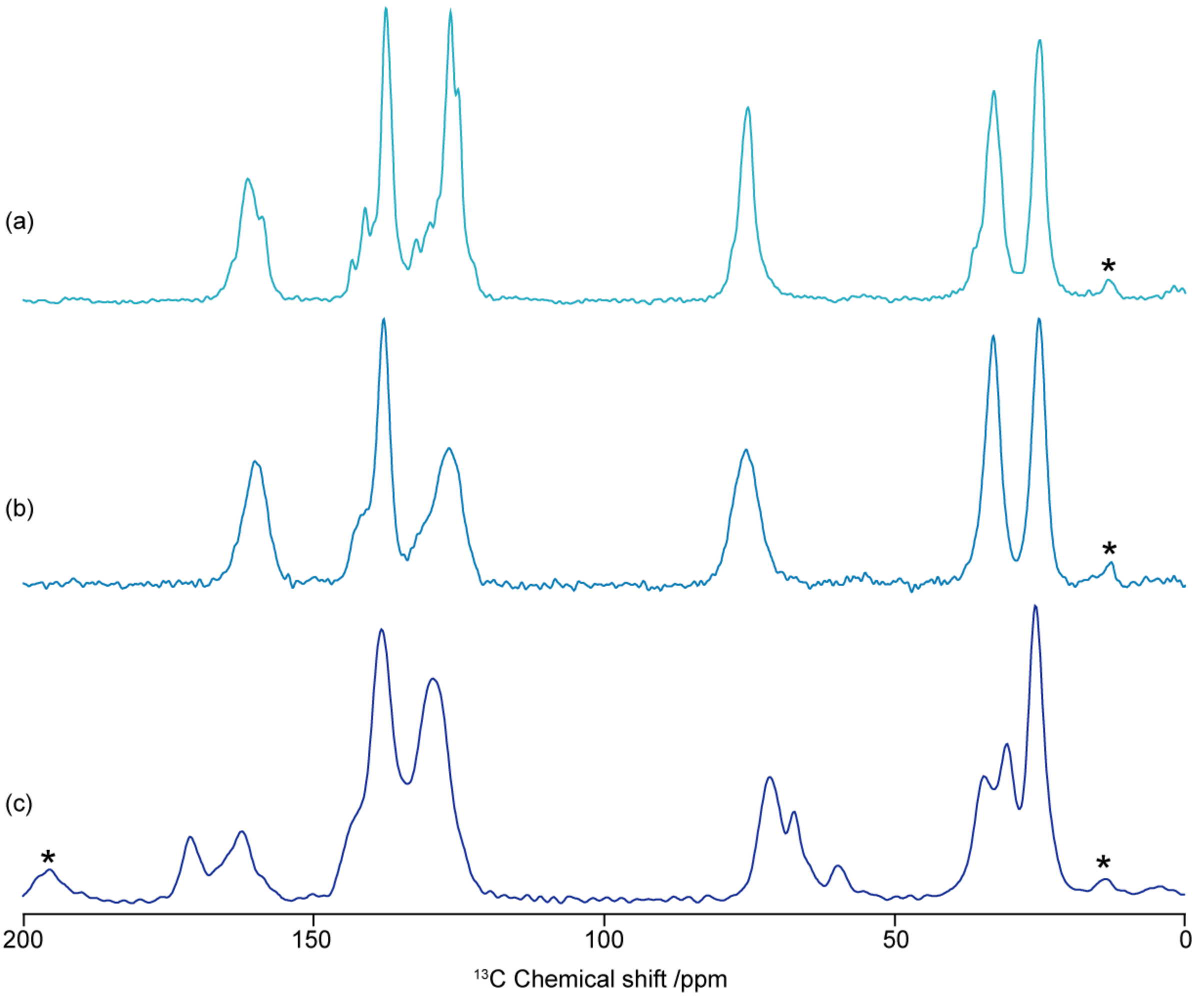

Figure S2. ^{13}C CP MAS NMR spectra at ν_r = 12.5 kHz of (a) TCC2-*R* (light blue), (b) [D_{12}]TCC2-*R* (blue) and (c) iodine-loaded TCC2-*R* (dark blue). Asterisks (*) denote spinning sidebands.

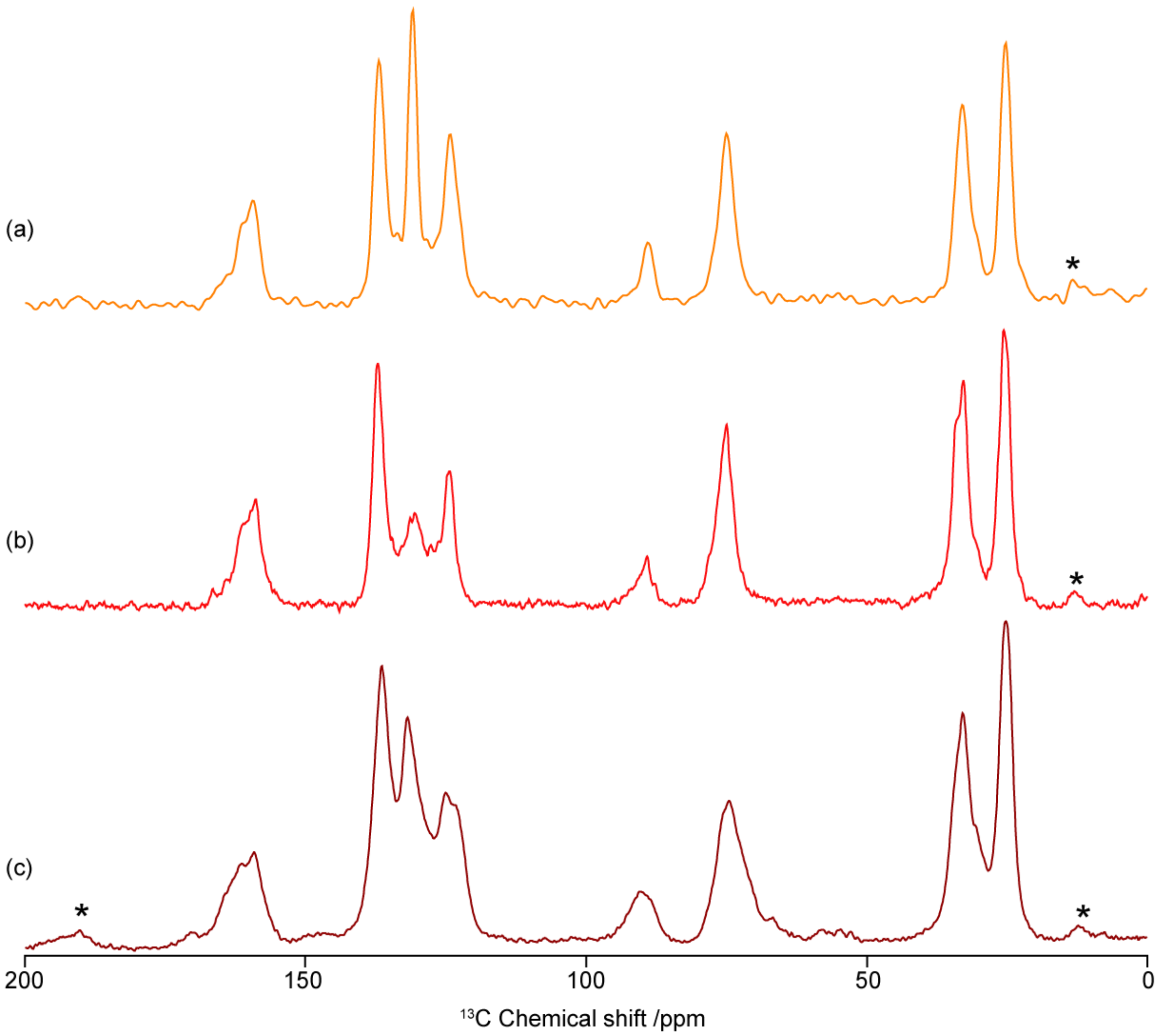

Figure S3. ^{13}C CP MAS NMR spectra at ν_r = 12.5 kHz of (a) TCC3-*R* (orange), (b) $[D_{12}]$TCC3-*R* (red) and (c) iodine-loaded TCC3-*R* (burgundy). Asterisks (*) denote spinning sidebands.

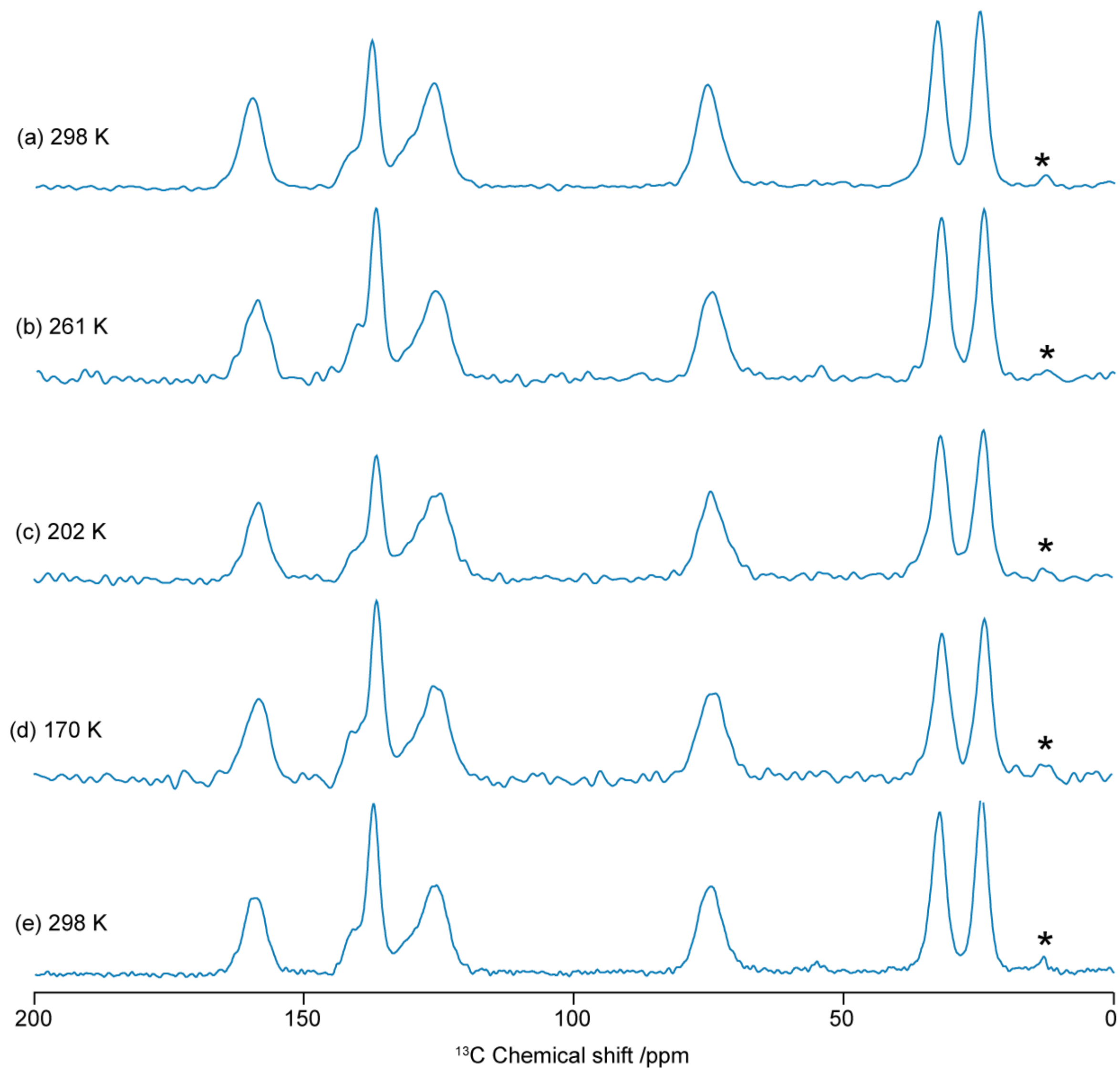

Figure S4. ^{13}C CP MAS NMR spectra at ν_r = 12.5 kHz of [D_{12}]TCC2-*R* at various temperatures. Spectrum (a) recorded at room temperature prior to cooling, followed by (b), (c) and (d) after equilibration at the corresponding temperatures. Spectrum (e) was recorded after warming back up to 298 K from 170 K. Asterisks (*) denote spinning sidebands.

Figure S4 shows that no change is observed in the ^{13}C CP MAS NMR spectra after cooling to low temperatures and heating back to room temperature.

Table S2. Comparison of the activation energies barrier (E_a), attempt frequencies (k_0) obtained from the Arrhenius equation (Figure 3), and change in enthalpy (ΔH) and entropy (ΔS) parameters derived from the Eyring equation for all the TCC cages investigated.

Tubular covalent cages	E_a /kJ mol^{-1}[a]	k_0 /Hz	ΔH /kJ mol^{-1}	ΔS /J K^{-1} mol^{-1}
[D_{12}]TCC2-*R*	18 (18-20)	(9 ± 4) x10^{10}	18 ± 1	-42 ± 5
Iodine-loaded [D_{12}]TCC2-*R*	21 (15-21)	(10 ± 8) x10^{9}	17 ± 1	-65 ± 11
[D_{12}]TCC3-*R*	12 (10-13)	(10 ± 7) x10^{9}	10 ± 3	-62 ± 7
Iodine-loaded [D_{12}]TCC3-*R*	21 (14-21)	(4 ± 4) x10^{9}	16 ± 3	-72 ± 12

[a] Range of E_a values estimated from errors in the values of k are given in brackets.

The change in enthalpy values shown in Table S2 are concordant with the activation energy data which show that the *para*-phenylene ring rotates more easily in [D_{12}]TCC3-*R* than in [D_{12}]TCC2-*R*. With iodine loading, [D_{12}]TCC3-*R* shows a strong increase in both its activation energy and ΔH values, validating that rotation has become more difficult. [D_{12}]TCC2-*R* does not show a drastic increase in enthalpy upon loading, indicating that iodine is possibly entering the voids within the lattice rather than the molecular pores. All ΔS are negative suggesting correlated motion leading to a relatively low attempt frequency k_0.[S8,S9]

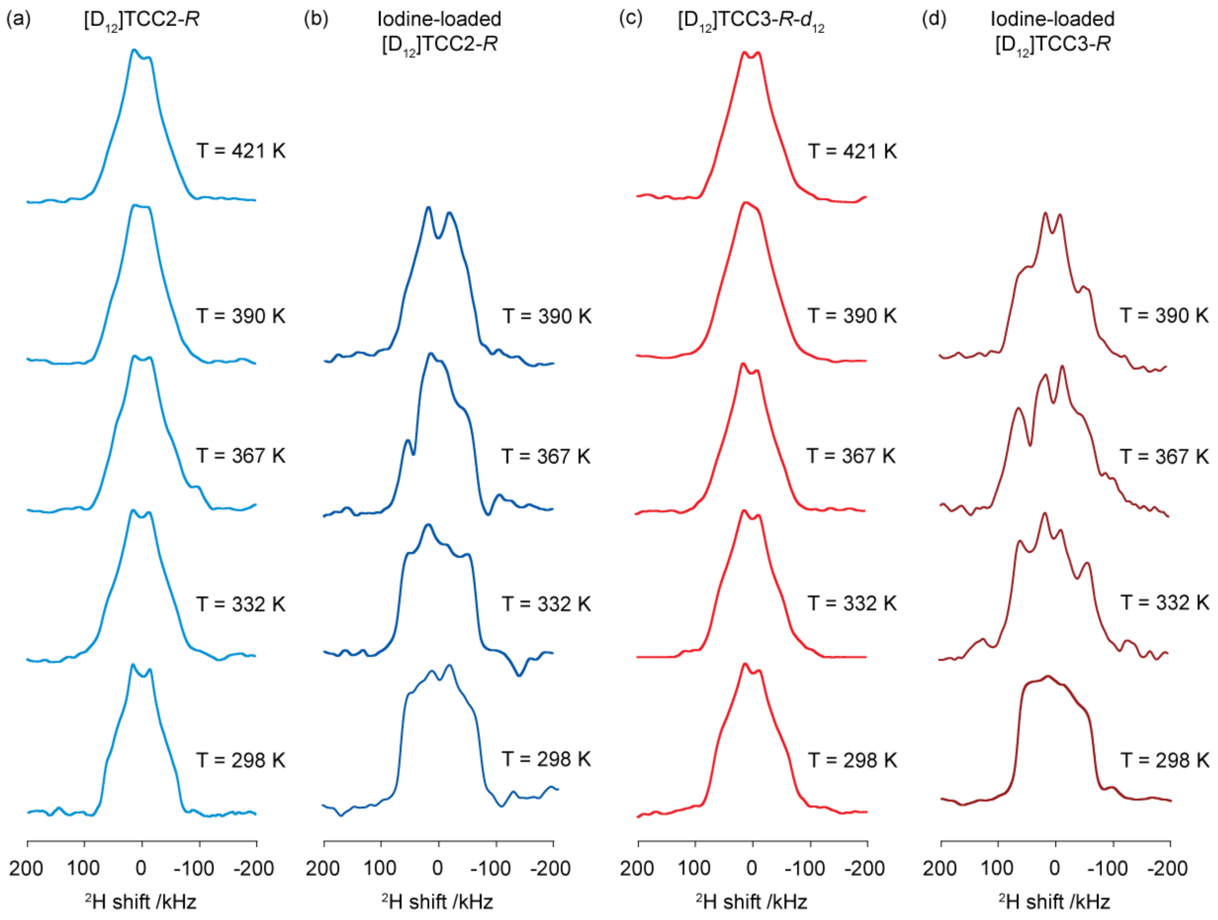

Figure S5. ^{2}H static solid echo NMR spectra of (a) [D$_{12}$]TCC2-*R* (blue), (b) iodine-loaded [D$_{12}$]TCC2-*R* (dark blue), (c) [D$_{12}$]TCC3-*R* (red), (d) iodine-loaded [D$_{12}$]TCC3-*R* (burgundy), obtained at temperatures of 298 K and above.

Above 298 K, the line shapes of the empty cages remains constant, being in the order of the fast motional regime. However, the iodine-loaded cages continue to exhibit lineshape narrowing at higher temperatures, implying faster motion, which is likely a result of iodine being released from the cages at these temperatures, as confirmed by thermogravimetric analysis below (Section S3).

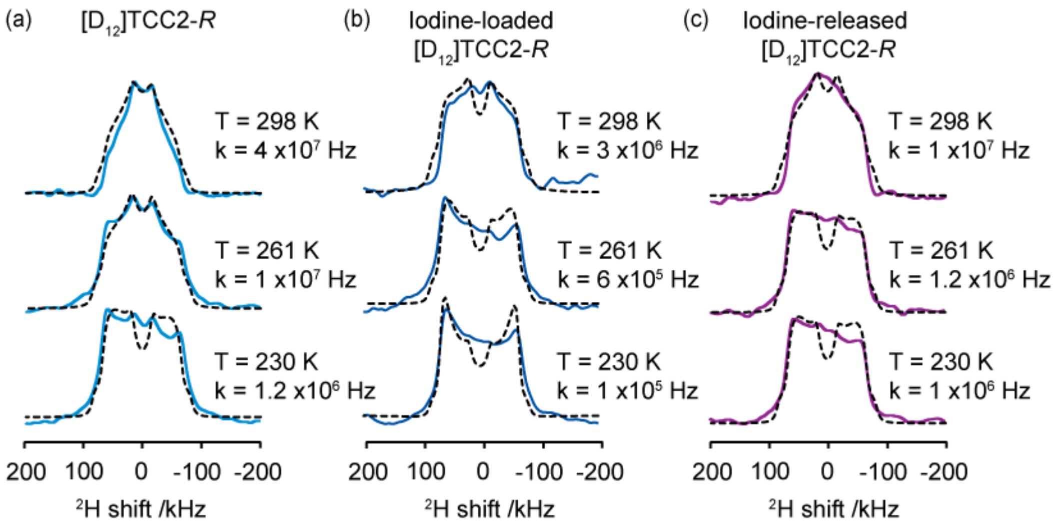

Figure S6. ^{2}H static solid echo NMR spectra of (a) [D_{12}]TCC2-*R* (blue), (b) iodine-loaded [D_{12}]TCC2-*R* (dark blue), (c) iodine-released [D_{12}]TCC2-*R* (purple), obtained at various temperatures. The rotational rates, k, obtained from numerical simulations of the NMR lineshapes are also given.

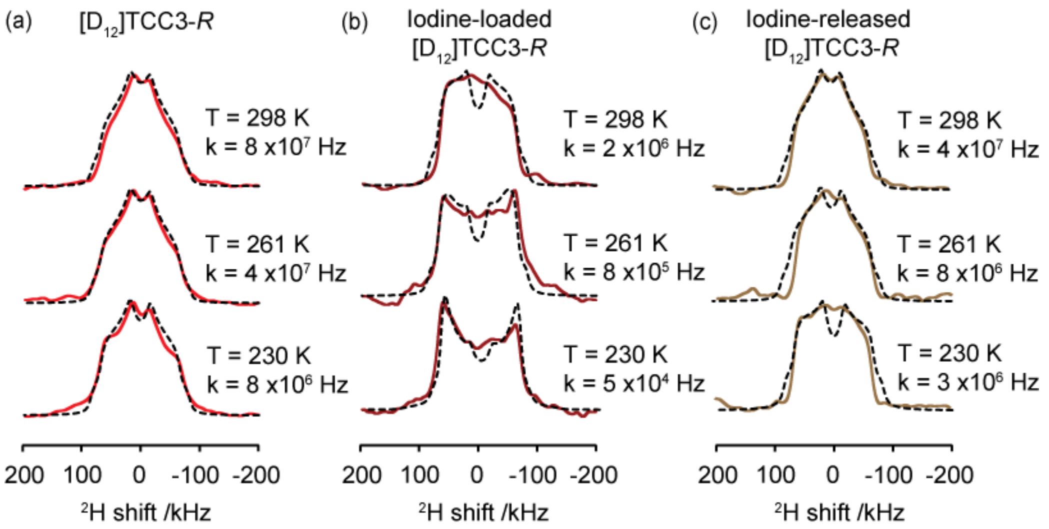

Figure S7. ^{2}H static solid echo NMR spectra of (a) [D_{12}]TCC3-*R* (red), (b) iodine-loaded [D_{12}]TCC3-*R* (burgundy), (c) iodine-released [D_{12}]TCC3-*R* (brown), obtained at various temperatures. The rotational rates, k, obtained from numerical simulations of the NMR lineshapes are also given.

After iodine-loaded [D_{12}]TCC2-*R* and [D_{12}]TCC3-*R* were heated for 7 hours at 70 °C, ^{2}H static solid echo NMR spectra were re-ran and their simulations obtained. These are shown below in Figures S6 and S7 for [D_{12}]TCC2-*R* and [D_{12}]TCC3-*R* respectively. It can be seen that after iodine has been released from the cages, rotational rates increase to close to that of the fast motional regime shown in the pristine cages (see Figure 2 in main text).

S2. Powder XRD data

Laboratory powder X-ray diffraction (PXRD) data were collected in transmission mode on samples held on thin Mylar film in aluminium well plates on a Panalytical X'Pert PRO MPD equipped with a high throughput screening (HTS) XYZ stage, X-ray focusing mirror, and PIXcel detector, using Ni-filtered Cu Kα radiation. Data were measured over the range 5−50° over 30 min.

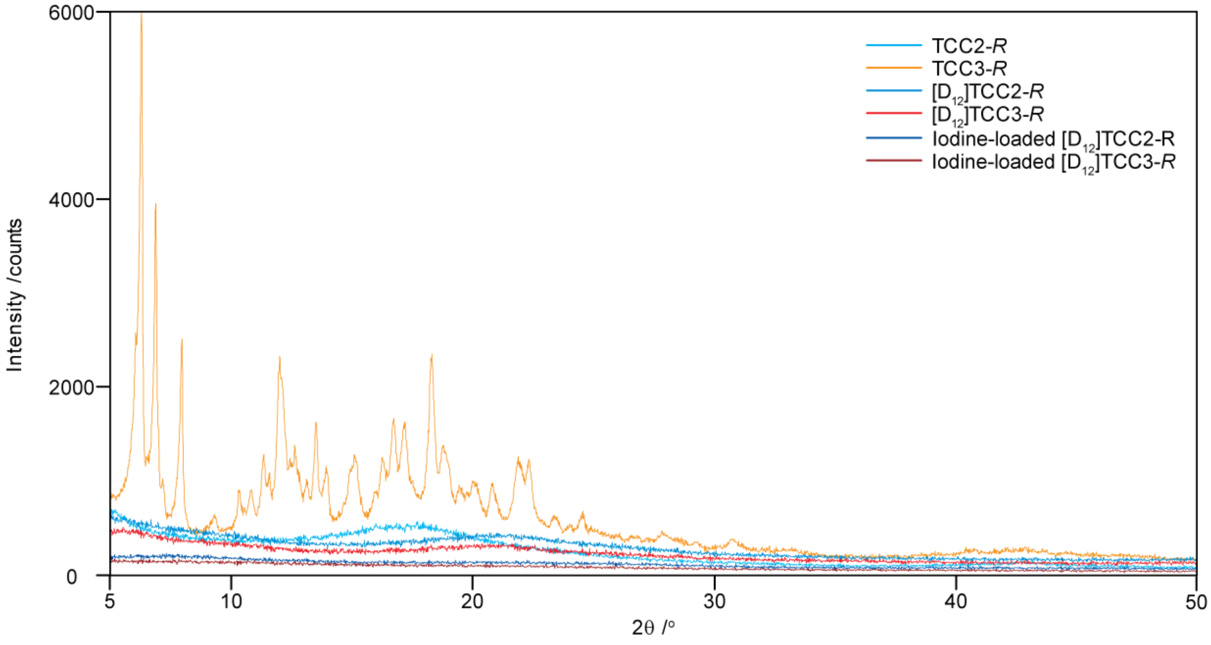

Figure S8. Powder XRD profiles of TCC2-R (light blue), TCC3-*R* (orange), $[D_{12}]$TCC2-*R* (blue), $[D_{12}]$TCC3-*R* (red), iodine-loaded $[D_{12}]$TCC2-*R* (dark blue) and iodine-loaded $[D_{12}]$TCC3-*R* (burgundy).

S3. Thermogravimetric Analysis

TGA analyses were carried out using a Q5000 IR analyzer (TA instruments). The samples were heated at the rate of 5 °C /min, being held for 10 minutes at 70 °C and 90 °C.

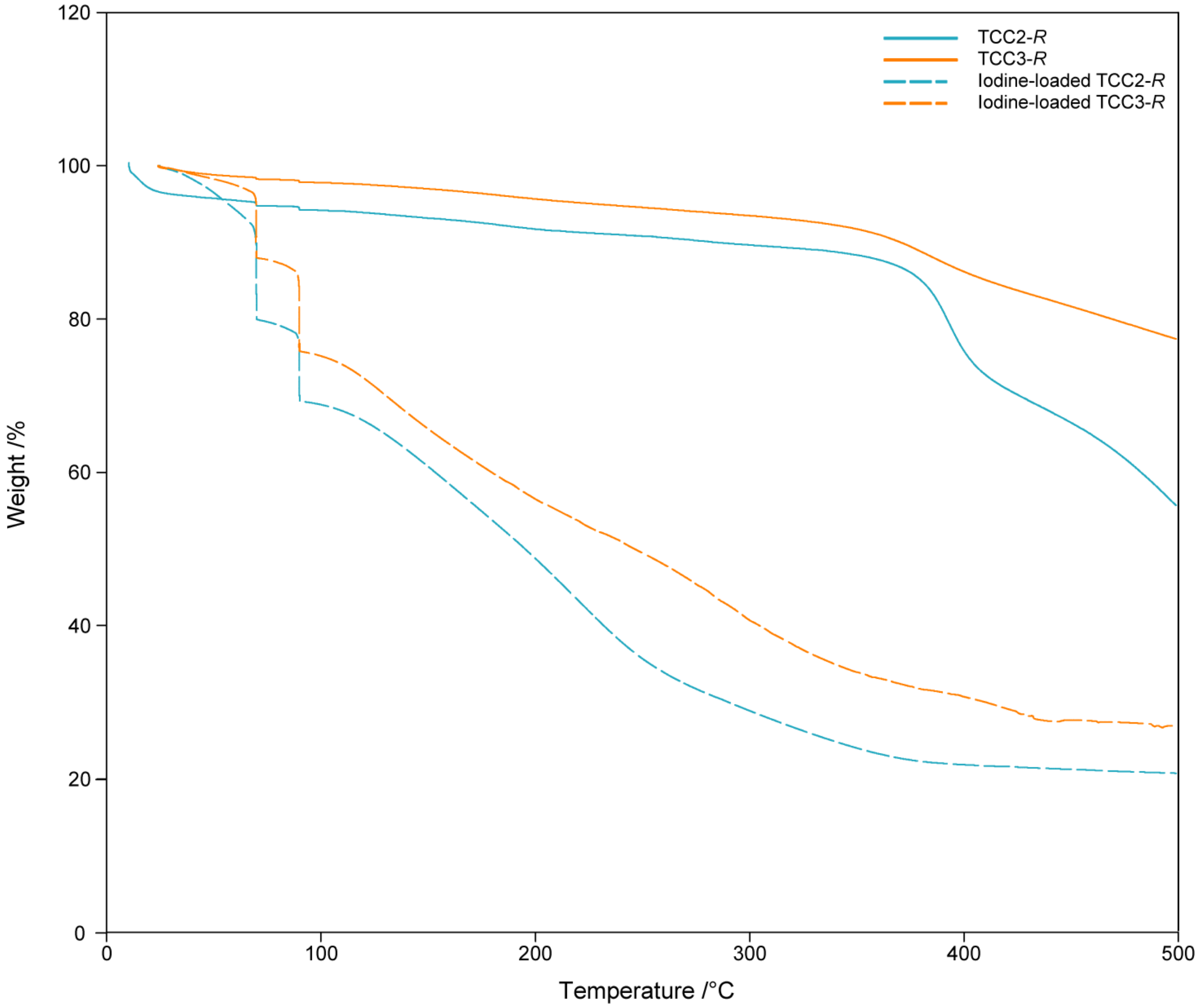

Figure S9. Thermogravimetric analysis of TCC2-*R* (light blue), TCC3-*R* (orange), iodine-loaded TCC2-*R* (dashed light blue) and iodine-loaded TCC3-*R* (dashed orange).

S4. Infrared Spectroscopy

Infrared spectra were collected on a Bruker ALPHA platinum attenuated total reflectance fourier transform infrared spectrometer (ATR-FTIR). Spectra were recorded for 24 scans in transmission mode.

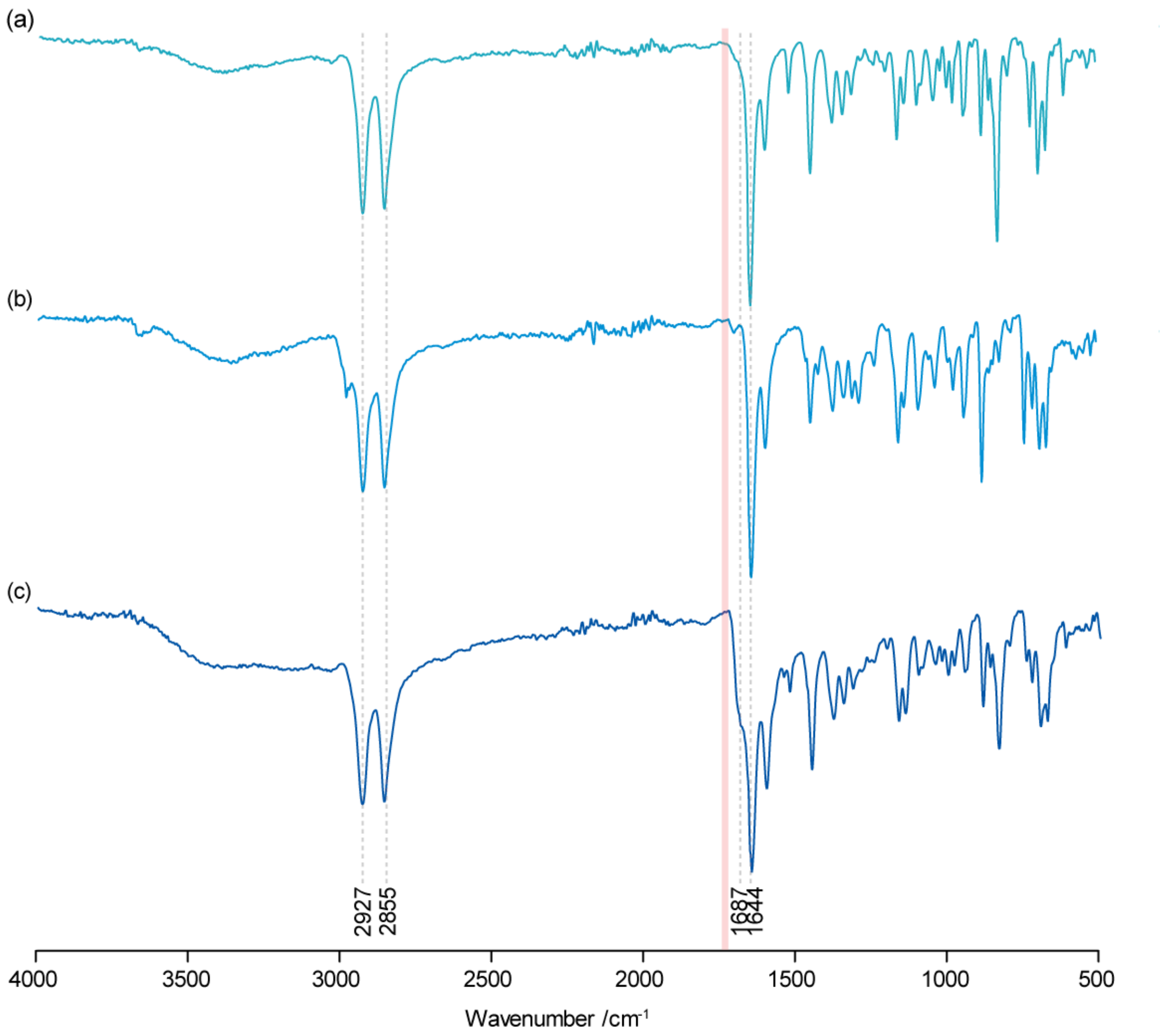

Figure S10. ATR-FTIR spectra of (a) TCC2-*R* (light blue), (b) [D_{12}]TCC2-*R* (blue) and (c) iodine-loaded [D_{12}]TCC2-*R* (dark blue). The red shaded region between 1720 and 1740 cm^{-1} and the dotted lines at 1687 and 1644 cm^{-1} are the anticipated area for the strong aldehyde carbonyl CO stretch[S10] and the known frequencies for imine stretching mode,[S10,S11] respectively.

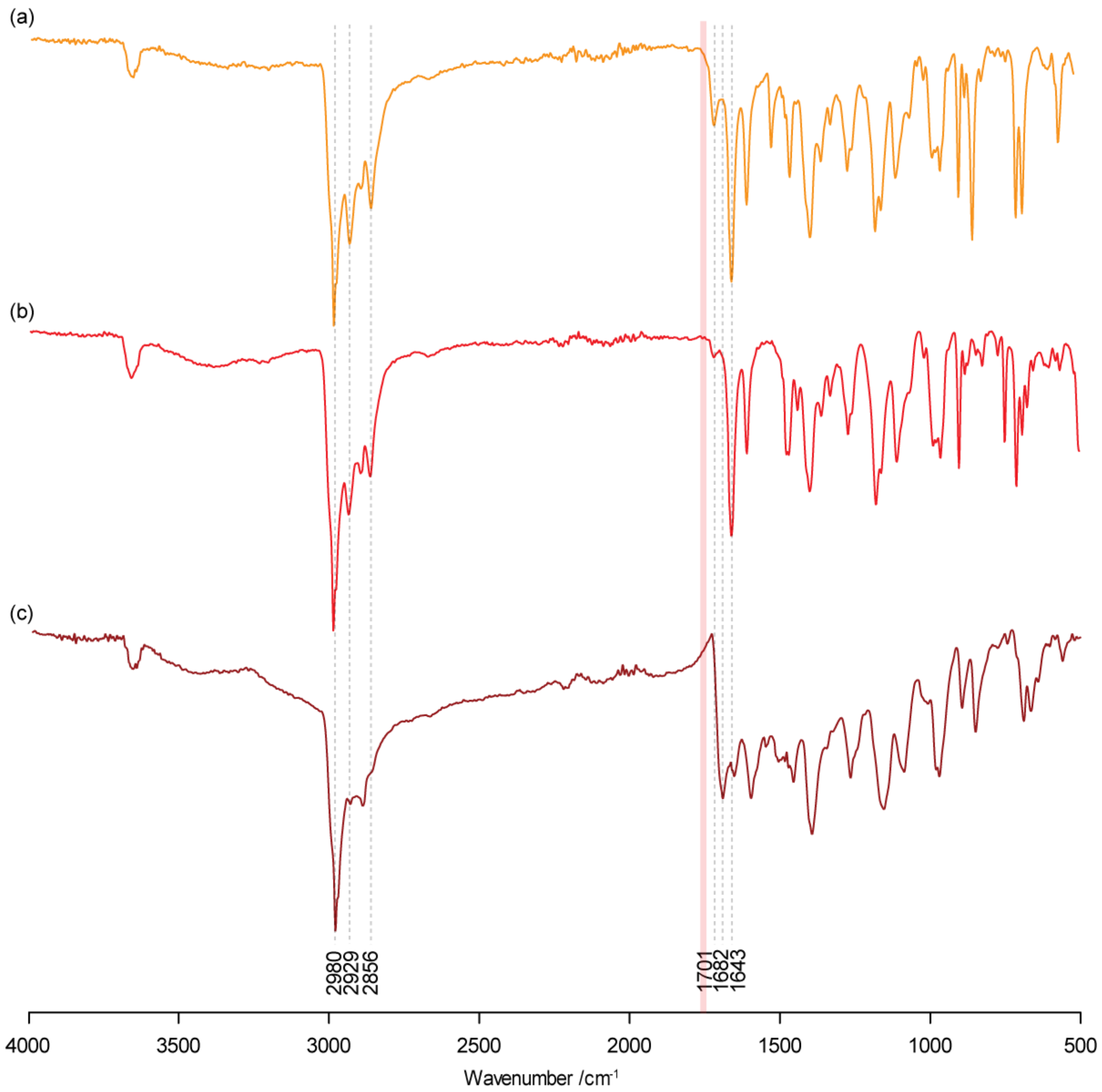

Figure S11. ATR-FTIR spectra of (a) TCC3-*R* (orange), (b) [D_{12}]TCC3-*R* (red) and (c) iodine-loaded [D_{12}]TCC3-*R* (burgundy). The red shaded region between 1720 and 1740 cm^{-1} and the dotted lines at 1682 and 1643 cm^{-1} are the anticipated area for the strong aldehyde carbonyl CO stretch[S10] and the known frequencies for imine stretching mode,[S10,S11] respectively. Flexibility within the TCC3-*R* structure[S12] allows for multiple imine modes to be visible.

S5. Syntheses of the chiral tubular covalent cages[S12]

The TCC2-*R* and TCC3-*R* cages were prepared accordingly to a literature procedure.[S11] The deuterated cages, $[D_{12}]$TCC2-*R* and $[D_{12}]$TCC3-*R* were prepared using the syntheses outlined below. 5-Bromoisophthalaldehyde was synthesized according to literature procedures.[S12,S13] All other chemicals were purchased from Sigma Aldrich or TCI and used as received.

S5.1. Synthesis of the aldehyde precursors

General synthesis consideration

Analytical HPLC analysis was conducting using a Dionex Ultimate 3000 HPLC system. Solution NMR spectra were recorded using either a Bruker Avance 400 MHz or 500 MHz NMR spectrometer. Mass spectrometry was carried out by the EPSRC National Mass Spectrometry Facility at Swansea University using a Xevo G2-S ASAP (QTOF), GCT Premier GC/MS (GC-EI-MS), or ultrafleXtreme (MALDI) instrument, and at the Microbiorefinary (University of Liverpool) using an Agilent Technologies 6530B accurate-mass QTOF Dual ESI mass spectrometer. Thermogravimetric analysis was carried out using a Q5000IR analyser (TA Instruments). IR spectra were recorded using a Bruker Tensor 27 FTIR spectrometer with Quest ATR attachment.

Synthesis of 2,2'-($[D_4]$1,4-phenylene)bis(4,4,5,5-tetramethyl-1,3,2-dioxaborolane) (1)

[1,1'-Bis(diphenylphosphino)ferrocene]dichloropalladium (II) (0.412 g, 0.563 mmol) was added to a degassed suspension of $[D_4]$1,4-dibromobenzene-2,3,5,6 (2.0 g, 8.3 mmol), bis(pinacolato)diboron (4.8 g, 19 mmol), and potassium acetate (5.2 g, 53 mmol) in 1,4-dioxane (40 mL) and the mixture was heated at reflux under a nitrogen atmosphere for 3 days. After cooling to ambient temperature, the reaction mixture was diluted with hexane (60 mL) and the suspension was charged to a pad of silica gel and washed with 1 % ethyl acetate/hexane until all the product had eluted. The filtrate was evaporated to dryness. The residue was redissolved in CH_2Cl_2, diluted with hexane and evaporated until ~20 mL of solvent remained. The suspension was filtered and the solid dried under vacuum to yield the desired pure compound **1** (2.0 g, 6.0 mmol, 75 %). ^1H NMR (500 MHz, $CDCl_3$): δ 1.35 (24 H, s, 8 x CH_3); ^{13}C NMR (101 MHz, $CDCl_3$): δ 133.59 (t, ^1J (C,D) = 24.3 Hz), 83.97, 25.01 ppm. MS(CI)$^+$ calcd for $C_{18}H_{25}D_4B_2O_4$ $[M+H]^+$: 335.1; found: 335.3. HRMS (TOF) calcd for $C_{18}H_{25}D_4B_2O_4$ $[M+H]^+$: 335.2503; found: 335.2511.

Synthesis of (($[D_4]$1,4-phenylene)bis(ethyne-2,1-diyl))bis(trimethylsilane) (2)

$[D_4]$1,4-dibromobenzene-2,3,5,6 (2.50 g, 10.4 mmol) was added to a flame-dried, backfilled flask. Et_3N (36 mL) was added via cannula. CuI (0.048 g, 0.25 mmol) and $Pd(PPh_3)_4$ (0.15 g, 0.13 mmol) were added, followed by the dropwise addition of trimethylsilyl acetylene (3.7 mL, 25 mmol). The mixture was refluxed for 8 hours, cooled, and then evaporated under reduced pressure to yield a crude off-white solid. This solid was purified via column chromatography (Biotage Isolera4, KP-Sil cartridge, 100 % hexane) to yield the product **2** as a white solid (2.6 g, 9.5 mmol, 95 %). ^1H NMR (400 MHz, $CDCl_3$ + TFA): δ 0.14 (18 H, s, CH_3); ^{13}C NMR (101 MHz, $CDCl_3$): δ 130.49 (t, ^1J (C,D) = 25.2 Hz), 103.64, 95.46, -0.96 ppm. MS(CI)$^+$ calcd for $C_{16}H_{19}D_4Si_2$ $[M+H]^+$: 275.5; found: 275.2. HRMS (TOF) calcd for $C_{16}H_{19}D_4Si_2$ $[M]^+$: 274.1511; found: 274.1516.

Synthesis of [D_4]1,4-diethynylbenzene-2,3,5,6 (3)

(([D_4]1,4-phenylene)bis(ethyne-2,1-diyl))bis(trimethylsilane) **2**, (2.5 g, 9.2 mmol) was dissolved in MeOH (50 mL) and CH_2Cl_2 (50 mL). K_2CO_3 (8.5 g, 62 mmol) was added, and the mixture stirred at room temperature for 24 hours. The colourless solution was then poured into H_2O (100 mL), extracted with Et_2O (3 x 100 mL) and washed with brine (200 mL). The organic layer was dried over $MgSO_4$, filtered, and evaporated under reduced pressure to yield the pure product **3** as a white solid (0.99 g, 7.6 mmol, 83 %). ^1H NMR (500 MHz, $CDCl_3$ + CF_3CO_2H): δ 3.17 (2 H, s, CH); ^{13}C NMR (101 MHz, $CDCl_3$): δ 130.76 (t, ^1J (C,D) = 25.0 Hz), 121.49, 82.11, 78.24 ppm. MS(CI)$^+$ calcd for $C_{10}H_3D_4$ [M+H]$^+$: 131.2; found: 131.1. HRMS (TOF) calcd for $C_{10}H_3D_4$ [M]$^+$: 130.0721; found: 130.0716.

Synthesis of [1,1':4',1"-terphenyl]-3,3"5,5"-tetracarbaldehyde (4)

5-Bromoisophthalaldehyde (1.9 g, 9 mmol), 2,2'-([D_4]1,4-phenylene)bis(4,4,5,5-tetramethyl-1,3,2-dioxaborolane) **1** (1.0 g, 3 mmol), and K_2CO_3 (4.1 g, 30 mmol) were suspended in $(CH_2)_4O$ (50 mL) and H_2O (15 mL) and thoroughly degassed with N_2 for 30 minutes. $Pd(PPh_3)_4$ (0.17 g, 0.15 mmol) was added and the mixture was heated at 85 °C under N_2 for 18 hours. A white precipitate was observed. The solution was then cooled to room temperature and the precipitate was collected by suction filtration. The filter cake was washed with Et_2O (100 mL), H_2O (100 mL), and a further portion of Et_2O (100 mL) and dried under suction to yield the desired pure compound **4** (0.89 g, 2.6 mmol, 87 %). ^1H NMR (400 MHz, $CDCl_3$ + CF_3CO_2H): δ 10.20 (4 H, s, CHO), 8.59 (4 H, d, ^4J = 1.6 Hz, Ar-H) 8.55 (2 H, t, ^4J = 1.5 Hz, Ar-H); ^{13}C NMR (101 MHz, $CDCl_3$): δ 194.94, 143.15, 138.38, 137.05, 134.89, 131.99, 127.95 (t, ^1J (C,D) = 24.7 Hz) ppm. MS(CI)$^+$ calcd for $C_{22}H_{11}D_4O_4$ [M+H]$^+$: 347.1; found: 347.1. HRMS (TOF) calcd for $C_{22}H_{11}D_4O_4$ [M+H]$^+$: 347.1221; found: 347.1220.

Synthesis of 1,4-bis(3,5-diformylphenyl)phenylethynylbenzene (5)

5-Bromoisophthalaldehyde (4.6 g, 22 mmol), [D_4]1,4-diethynylbenzene-2,3,5,6 (**3**) (0.99 g, 7.6 mmol), and CuI (0.05 g, 0.26 mmol) were added to a flame-dried flask which had been backfilled with N_2 three times. Et_3N (distilled, 100 mL) was added, and the mixture was degassed with N_2 for 30 minutes. $Pd(PPh_3)_4$ (0.30 g, 0.26 mmol) was then added and the mixture was heated at 60 °C for 24 hours under N_2. The resulting suspension was diluted with water and the precipitate collected by filtration. The filter cake was washed with water and acetone, then dried under suction. The filter cake was transferred to a Soxhlet and extracted with CH_2Cl_2 for 24 h. The CH_2Cl_2 extracts were concentrated to ~50 mL, the suspension was filtered and the filter cake was washed with acetone and dried under suction to afford a yellow powder (1.9 g, 4.8 mmol, 63 %). ^1H NMR (500 MHz, $CDCl_3$): δ 10.11 (4 H, s, CHO), 8.43 (2 H, t, ^4J = 1.6 Hz, Ar-H), 8.37 (4 H, d, ^4J = 1.6 Hz, Ar-H). ^{13}C NMR (101 MHz, $CDCl_3$): δ 191.96, 137.57, 135.80, 130.75 (t, ^1J (C,D) = 24.4 Hz), 130.01, 125.25, 121.74, 91.65, 87.47. ppm. MS(CI)$^+$ calcd for $C_{26}H_{11}D_4O_4$ [M+H]$^+$: 395.4; found: 395.1. HRMS (TOF) calcd for $C_{26}H_{11}D_4O_4$ [M+H]$^+$: 395.1221; found: 395.1219.

S5.2. Synthesis of the chiral tubular covalent cages

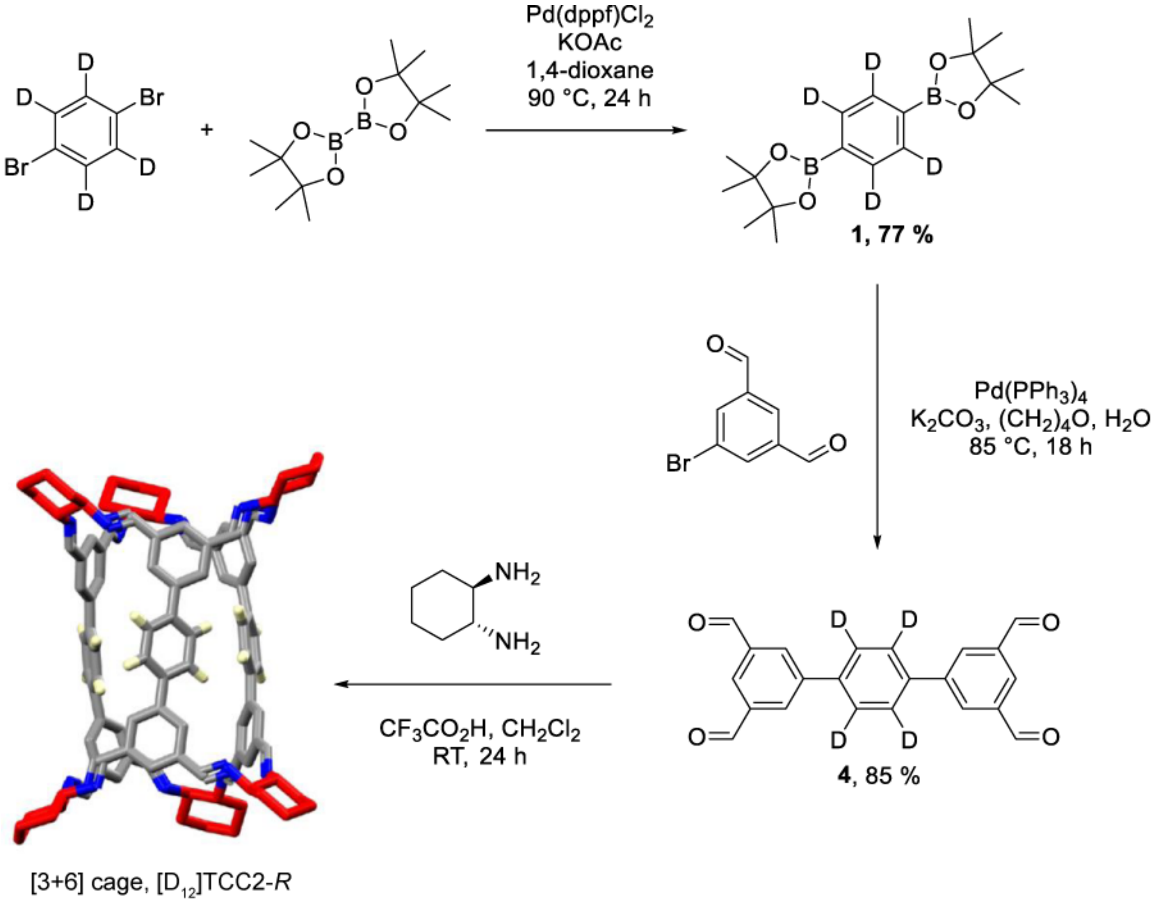

Scheme S1. Synthesis of cage compound [D_{12}]TCC2-*R* from the deuterated precursor **1** and aldehyde **4** via a palladium catalysed coupling reaction. The cyclohexane groups are shown in red; other C, grey; N, blue; D, yellow; H, omitted for clarity in the crystal structure representation.

Synthesis of solvated cage compound [D_{12}]TCC2-*R*

To a stirred suspension of **4** (0.80 g, 2.3 mmol) and CF_3CO_2H (2 drops) in CH_2Cl_2 (15 mL) was added a solution of *R*,*R*-cyclohexanediamine (0.53 g, 4.6 mmol) in CH_2Cl_2 (10 mL). The mixture was stirred overnight at room temperature, during which time the solution turned yellow and the tetraaldehyde compound was seen to dissolve. After five days, the reaction mixture was diluted with CH_2Cl_2 and the mixture was filtered to remove any insoluble byproducts. The filtrate was concentrated to ~20 mL, hexane (40 mL) was charged with stirring and the resulting white precipitate was collected via suction filtration to yield [D_{12}]TCC2-*R* (0.88 g, which contains 1.3 wt% hexane as calculated by integration of additional solvent peaks in the NMR at 1.26 and 0.83 ppm, adjusted yield 0.87 g, 0.58 mmol, 75 %). ^{1}H NMR (400 MHz, CD_3OD + $CDCl_3$): δ 8.28 (6 H, s), 8.19 (6 H, s), 7.88 (12 H, m), 7.41 (6 H, s), 3.50 (6 H, br. m.), 3.29 (6 H, m), 2.10 – 2.03 (6 H, m), 1.98 – 1.76 (30 H, m), 1.55 (12 H, m). ^{13}C NMR (101 MHz, $CDCl_3$): δ 162.21, 161.87, 141.21, 138.97, 136.55, 135.84, 131.99, 126.77, 125.93, 125.57, 75.20, 73.90, 32.20, 31.58, 24.26, 24.23. HRMS (TOF) calcd for $C_{102}H_{92}D_{12}N_{12}$ $[M+2H]^{2+}$: 754.4630; found: 754.4659 $[M+2H]^{2+}$ and 503.3142 $[M+3H]^{3+}$.

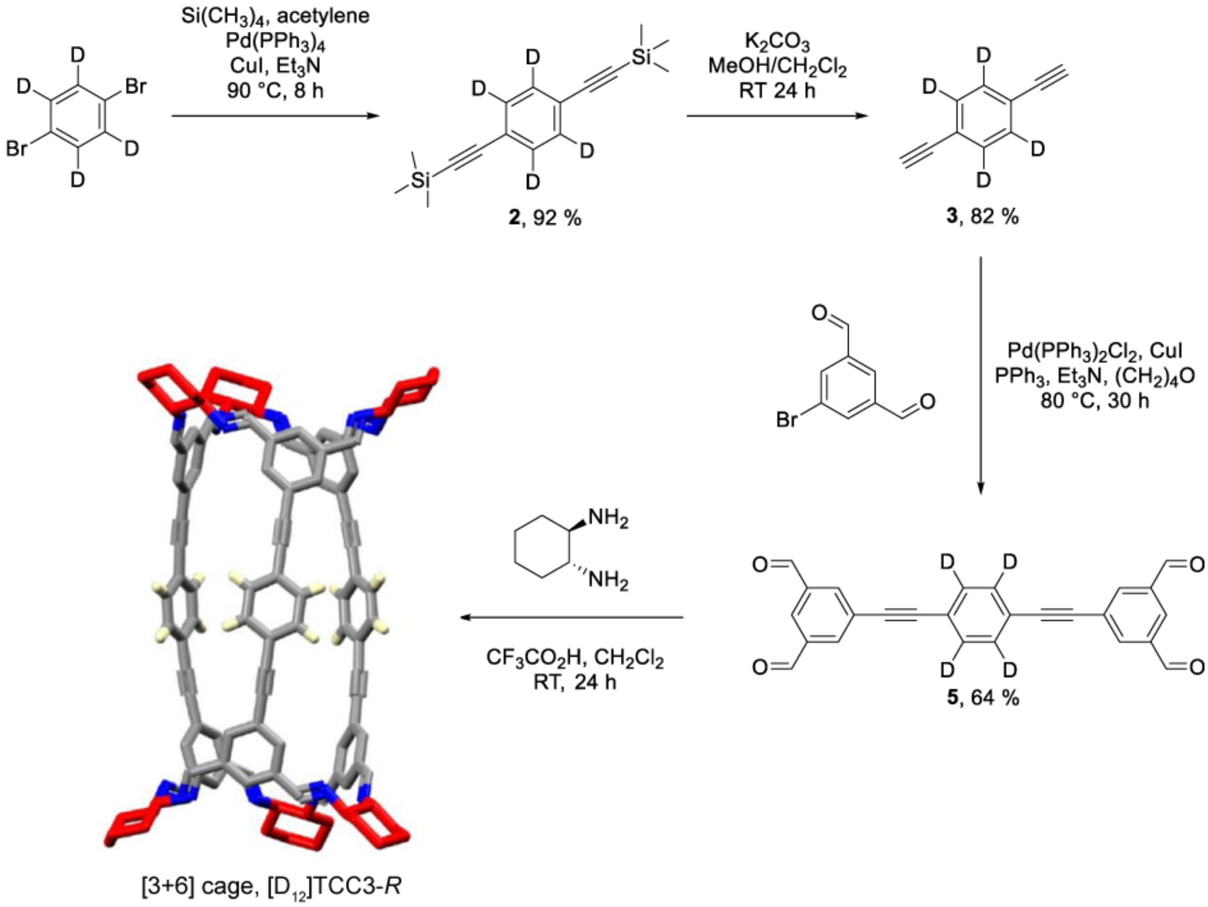

Scheme S2. Synthesis of cage compound [D_{12}]TCC3-*R* from the deuterated precursor **3** and aldehyde **5** via a palladium catalysed coupling reaction. The cyclohexane groups are shown in red; other C, grey; N, blue; D, yellow; H, omitted for clarity in the crystal structure representation.

Synthesis cage compound [D_{12}]TCC3-*R*

[D_{12}]TCC3-*R* was prepared using the same as for [D_{12}]TCC2-*R* method above using aldehyde **5**, and required repeated recrystallisations from CH_2Cl_2/hexane to obtain a pure product (0.40 g from 1.00 g aldehyde, no hexane observed in the ^{1}H NMR, 0.24 mmol, 28 %). ^{1}H NMR (500 MHz, MeOD/$CDCl_3$): δ 8.17 (6 H, s, N=CH), 8.16 (6 H, s, N=CH), 7.95 (6 H, m, Ar-H), 7.89 (6 H, m, Ar-H), 7.50 (6 H, m, Ar-H), 3.45 (6 H, m, NCH), 3.37 (6 H, m, NCH), 1.90–1.50 (48 H, m, CH_2) ^{13}C NMR (101 MHz, $CDCl_3$): δ 160.37, 160.05, 136.6, 136.2, 135.9, 131.05 (t, ^1J (C,D) = 26.1 Hz), 129.64, 127.58, 123.91, 122.62, 90.27, 90.06, 75.04, 74.30, 32.71, 32.35, 24.2, 24.15. HRMS (TOF) calcd for $C_{114}H_{90}D_{12}N_{12}$ $[M+2H]^{2+}$: 826.4630; found: 826.4674 $[M+2H]^{2+}$ and 551.3152 $[M+3H]^{3+}$.

S5.3. Preparation of all the desolvated cages

Prior to solid state NMR, all pristine cages were dried at 333 K for 24 hours under vacuum to ensure no residual solvent or water was present.

S5.4. Iodine loading into the cages[S14]

The cages were initially desolvated under vacuum and pristine TCC2-*R*, TCC3-*R*, [D_{12}]TCC2-*R* and [D_{12}]TCC3-*R* (*ca*. 0.05-0.11 g, 0.03-0.07 mmol) were held in a non-porous aluminum cup in an atmosphere of I_2 in large excess (~1 g, ~4 mmol, ~60-130 equiv.) at atmospheric pressure. The uptake of iodine was monitored gravimetrically as shown in Figure S12(a) below and the white [D_{12}]TCC2-*R* and pale yellow [D_{12}]TCC3-*R* cages became dark yellow within an hour and turned brown within a few hours (Figure S12 (b) and (c)), respectively. It was assumed that all weight increases were related only to the uptake of iodine into the cages. All NMR spectra were recorded after samples had been in an I_2 atmosphere for a minimum of 48 hours.

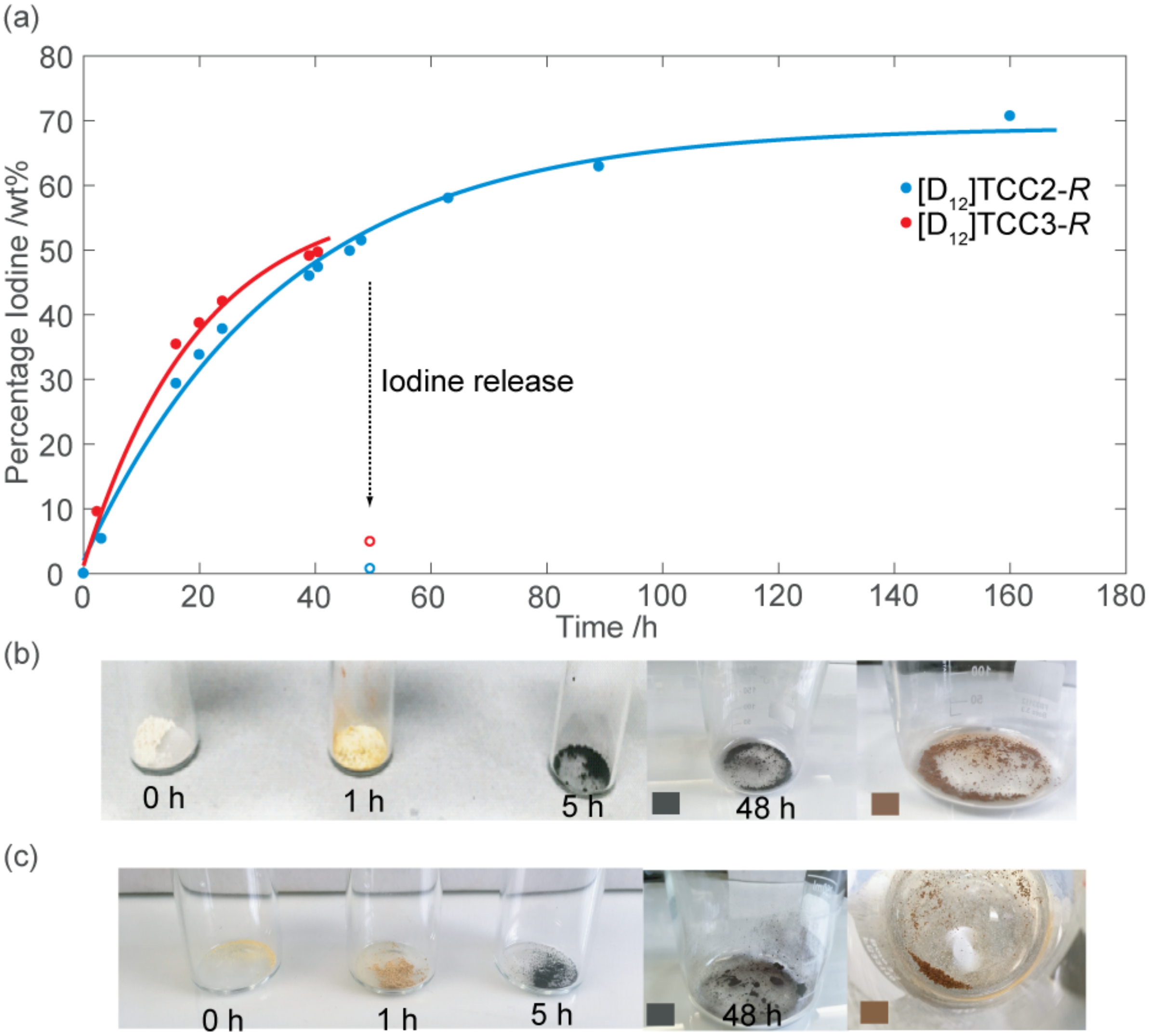

Figure S12. (a) Gravimetric uptake of iodine into [D_{12}]TCC2-*R* (blue) and [D_{12}]TCC3-*R* (red) cages as a function of time at room temperature. Empty circles show the percentage weight of iodine left after heating the [D_{12}]TCC2-*R* and [D_{12}]TCC3-*R* samples for 7 hours at 70 °C. Photographs showing the colour change of (b) [D_{12}]TCC2-*R* and (c) [D_{12}]TCC3-*R* during iodine uptake after 1, 5 and 48 hours with the picture on the right showing the iodine-released samples after 7 hours at 70 °C.

After 40.5 hours, [D_{12}]TCC2-*R* (0.110 g, 0.073 mmol) had absorbed 0.090 g of iodine atoms (0.709 mmol), resulting in an uptake of 10 iodine atoms per molecule. After 40.5 hours, [D_{12}]TCC3-*R* (0.049 g, 0.030 mmol) had absorbed 0.044 g of iodine atoms (0.346 mmol), resulting in an uptake of 12 iodine atoms per molecule. The increase in weight is due to insertion of iodine into the void, however during the shallower section of the uptake (> 60 hours) for [D_{12}]TCC2-*R*, it is possible that the iodine is also interacting with the cage structure. Figures S2 and S10-S11 do not display ^{13}C CP MAS NMR or IR peak within the aldehyde carbonyl region (178-200 ppm and 1720-1740 cm^{-1}, respectively),[S10] indicating that no decomposition of the solid cages is occurring during iodine loading. The shoulder observed in the iodine-loaded [D_{12}]TCC2-*R* IR spectrum in Figure S10c at 1687 cm^{-1} is within the imine stretching region[S10] and probably indicates that charge transfer is occurring within this cage as previously observed on the tetrahedral organic cage molecule CC3[S15] from IR literature data.[S10-S11]

Upon heating for 7 hours at 70 °C, iodine was released from the cages with the colour changing significantly from dark brown/black to light brown as seen in Figure S12. Additionally, the mass decreased with a loss of 49 and 44 wt% of iodine for [D_{12}]TCC2-*R* and [D_{12}]TCC3-*R*, respectively, corresponding to almost complete release of iodine over one cycle.

S6. Rotational rates within the literature

Table S3. Comparison of molecular rotors molecular rotors with rotational frequencies of a a 180° site reorientation exceeding 10^5 Hz at selected temperature monitored by ^{2}H NMR within literature.

Molecular Rotor	Temperature /K	k /Hz	Ref
Porous Organic Materials			
[D_{12}]TCC2-*R*	298	4 x10^7	This work
	230	1.2 x10^6	
[D_{12}]TCC3-*R*	298	8 x10^7	This work
	230	8 x10^6	
	202	4 x10^6	
	180	3 x10^6	
Bis(sulfophenylethynyl)-benzene frameworks	290	1 x 10^8	[S17]
	200	1.8 x10^6	
	179	2 x10^5	
PAF3 [D_4]polytetraphenylsilane-based	300	1 x10^8	[S18]
	230 [a]	1.6 x10^6	
1,4-naphthalenediyl-bridged molecular gyrotops (C_{18}) [D_4]phenylene	290	1 x10^7	[S19]
	230 [a]	4.8 x10^6	
Porous molecular crystal with 4,4′-bis(sulfophenylethynyl) [D_4]benzene structure	295	1 x10^8	[S20]
	230	1 x10^6	
	193	5.6 x10^4	
Metal Organic Frameworks [b]			
Zn-1,4-bis(1*H*-pyrazol-4-ylethynyl)-[D_4]benzene	290	> 1 x10^8	[S21]
	150	> 1 x10^8	
Zn-interdigitated structure (x=0.55) [Zn(5-NO_2-ip)$_{0.55}$(5-MeO-ip)$_{0.45}$([D_8]bpy)] (DMF.MeOH)$_n$ (5-NO_2-ip = 5-nitroisophthalate, 5-MeO-ip = 5-methoxyisophthalate, [D_8]bpy = deuterated 4,4’-bipyridyl)	298	~ 5 x10^7	[S22]
Zr-[D_4]UiO66	296	2.3 x10^6	[S23]
Non Porous Crystalline Materials			
Co-crystal between tritylacetylene bromide TrBr and diazabicyclo[2.2.2] [D_{12}]octane ([D_{12}]dabco)	296	> 1 x10^8	[S24]
	150	> 1 x10^8	

[a] No data below 230 K are available. [b] Selection of fastest rotational frequencies of MOFs taken from within the literature.[S21]

S7. References

[S1] B. M. Fung, A. K. Khitrin, K. Ermolaev, *J. Magn. Reson.* **2000**, *142*, 97–101.

[S2] R. E. Ernst, G. Bodenhausen, A. Wokaun, *Principles of Nuclear Magnetic Resonance in One and Two Dimensions*, Oxford University Press, Oxford, **1987**.

[S3] The MathWorks Inc, **2016**.

[S4] P. A. Beckmann, C. Dybowski, *J. Magn. Reson.* **2000**, *146*, 379–380.

[S5] A. Bielecki, D. P. Burum, *J. Magn. Reson.* **1995**, *116*, 215–220.

[S6] C. R. Morcombe, K. W. Zilm, *J. Magn. Reson.* **2003**, *162*, 479–486.

[S7] R. L. Vold, G. L. Hoatson, *J. Magn. Reson.* **2009**, *198*, 57–72.

[S8] B. Rodríguez-Molina, N. Farfán, M. Romero, J. M. Méndez-Stivalet, R. Santillan, M. A. Garcia-Garibay, *J. Am. Chem. Soc.* **2011**, *133*, 7280–7283.

[S9] C. Lemouchi, K. Iliopoulos, L. Zorina, S. Simonov, P. Wzietek, T. Cauchy, A. Rodríguez-Fortea, E. Canadell, J. Kaleta, J. Michl, D. Gindre, M. Chrysos, P. Batail, *J. Am. Chem. Soc.* **2013**, *135*, 9366–9376.

[S10] R. M. Silverstein, F. X. Webster, D. J. Kiemle, *The Spectrometric Identification of Organic Compounds*, John Wiley & Sons, New York, **2005**.

[S11] F. R. Diaz, J. Moreno, L. H. Tagle, G. A. East, D. Radic, *Synth. Met.* **1999**, *100*, 187–193.

[S12] A. G. Slater, M. A. Little, A. Pulido, S. Y. Chong, D. Holden, L. Chen, C. Morgan, X. Wu, G. Cheng, R. Clowes, M. E. Briggs, T. Hasell, K. E. Jelfs, G. M. Day, A. I. Cooper, *Nat. Chem.* **2017**, *9*, 17–25.

[S13] O. A. Blackburn, B. J. Coe, M. Helliwell, J. Raftery, *Organometallics* **2012**, *31*, 5307–5320.

[S14] M. Wang, C. Wang, X.-Q. Hao, J. Liu, X. Li, C. Xu, A. Lopez, L. Sun, M.-P. Song, H.-B. Yang, X. Li, *J. Am. Chem. Soc.* **2014**, *136*, 6664–6671.

[S15] T. Hasell, M. Schmidtmann, A. I. Cooper, *J. Am. Chem. Soc.* **2011**, *133*, 14920–14923.

[S16] S. Bracco, T. Miyano, M. Negroni, I. Bassanetti, L. Marchio', P. Sozzani, N. Tohnai, A. Comotti, *Chem. Commun.* **2017**, *53*, 7776–7779.

[S17] A. Comotti, S. Bracco, T. Ben, S. Qiu, P. Sozzani, *Angew. Chemie Int. Ed.* **2014**, *53*, 1043–1047.

[S18] W. Setaka, K. Inoue, S. Higa, S. Yoshigai, H. Kono, K. Yamaguchi, *J. Org. Chem.* **2014**, *79*, 8288–8295.

[S19] A. Comotti, S. Bracco, A. Yamamoto, M. Beretta, T. Hirukawa, N. Tohnai, M. Miyata, P. Sozzani, *J. Am. Chem. Soc.* **2014**, *136*, 618–621.

[S20] A. Comotti, S. Bracco, F. Castiglioni, S. Galli, M. Negroni, A. Maspero, P. Sozzani, *Chem. A Eur. J.* **2017**, *23*, 11210–11215.

[S21] M. Inukai, T. Fukushima, Y. Hijikata, N. Ogiwara, S. Horike, S. Kitagawa, *J. Am. Chem. Soc.* **2015**, 12183–12186.

[S22] D. I. Kolokolov, A. G. Stepanov, V. Guillerm, C. Serre, B. Frick, H. Jobic, *J. Phys. Chem. C* **2012**, *116*, 12131–12136.

[S23] L. Catalano, S. Perez-Estrada, H.-H. Wang, A. J. L. Ayitou, S. I. Khan, G. Terraneo, P. Metrangolo, S. Brown, M. A. Garcia-Garibay, *J. Am. Chem. Soc.* **2017**, *139*, 843–848.
